# Recording of Electrically Evoked Neural Activity and Bladder Pressure Responses in Awake Rats Chronically Implanted With a Pelvic Nerve Array

**DOI:** 10.3389/fnins.2020.619275

**Published:** 2020-12-17

**Authors:** Sophie C. Payne, Nicole M. Wiedmann, Calvin D. Eiber, Agnes W. Wong, Philipp Senn, Peregrine B. Osborne, Janet R. Keast, James B. Fallon

**Affiliations:** ^1^Bionics Institute, Fitzroy, VIC, Australia; ^2^Medical Bionics Department, University of Melbourne, Melbourne, VIC, Australia; ^3^Department of Anatomy and Neuroscience, University of Melbourne, Melbourne, VIC, Australia; ^4^Department of Otolaryngology, University of Melbourne, Melbourne, VIC, Australia

**Keywords:** micturition, continence, splanchnic nerve, neuromodulation, pelvic ganglion

## Abstract

Bioelectronic medical devices are well established and widely used in the treatment of urological dysfunction. Approved targets include the sacral S3 spinal root and posterior tibial nerve, but an alternate target is the group of pelvic splanchnic nerves, as these contain sacral visceral sensory and autonomic motor pathways that coordinate storage and voiding functions of the bladder. Here, we developed a device suitable for long-term use in an awake rat model to study electrical neuromodulation of the pelvic nerve (homolog of the human pelvic splanchnic nerves). In male Sprague-Dawley rats, custom planar four-electrode arrays were implanted over the distal end of the pelvic nerve, close to the major pelvic ganglion. Electrically evoked compound action potentials (ECAPs) were reliably detected under anesthesia and in chronically implanted, awake rats up to 8 weeks post-surgery. ECAP waveforms showed three peaks, with latencies that suggested electrical stimulation activated several subpopulations of myelinated A-fiber and unmyelinated C-fiber axons. Chronic implantation of the array did not impact on voiding evoked in awake rats by continuous cystometry, where void parameters were comparable to those published in naïve rats. Electrical stimulation with chronically implanted arrays also induced two classes of bladder pressure responses detected by continuous flow cystometry in awake rats: voiding contractions and non-voiding contractions. No evidence of tissue pathology produced by chronically implanted arrays was detected by immunohistochemical visualization of markers for neuronal injury or noxious spinal cord activation. These results demonstrate a rat pelvic nerve electrode array that can be used for preclinical development of closed loop neuromodulation devices targeting the pelvic nerve as a therapy for neuro-urological dysfunction.

## Introduction

Electrical neuromodulation (neurostimulation) is an effective therapeutic technology for treating lower urinary tract (LUT) dysfunction in some patients. The United States Food and Drug Administration (FDA) has approved two targets—the sacral S3 spinal root and posterior tibial nerve (de Groat and Tai, 2018)—which are now well established, with commercial devices widely used to deliver safe, effective therapy. However, neither is effective across the full range of urological indications, including many neuro-urological conditions caused by dysfunction in the neural circuit that controls normal storage and voiding (micturition) and other urological functions.

Preclinical studies in animal models suggest that the pelvic nerve (rodent homolog of human pelvic splanchnic nerves) is a potential neuromodulation target for neuro-urological and other pelvic functional disorders (Crook and Lovick, 2017; Langdale et al., 2017; Crook et al., 2018; Langdale et al., 2020). For example, in cats and rodents, these paired nerves contain the majority of external sacral sensory and autonomic motor projections needed for bladder sensation and contraction. More specifically, they contain all LUT visceral sensory axons projecting from sacral dorsal root ganglia to the bladder and urethra, and all sacral spinal preganglionic axons that innervate postganglionic neurons in pelvic ganglia (inferior hypogastric plexus in human). Pelvic ganglia provide the parasympathetic motor innervation of the bladder and urethra smooth muscle. The predominance of visceral LUT afferents and autonomic efferents differentiates this pelvic nerve pathway as a neuromodulation target from existing devices (i.e., sacral, tibial, and pudendal nerve stimulation; transcutaneous stimulation of pudendal nerve or foot; Goldman et al., 2008; Yoo et al., 2009; Peters et al., 2010; Opisso et al., 2013; Ammi et al., 2014; Chen et al., 2014), which instead generally target somatic nerves and their central circuits. On this basis, electrical neuromodulation of pelvic nerves can be compared to vagal neuromodulation used to primarily target autonomic preganglionic efferent and visceral afferent axons.

Targeting pelvic nerve neuromodulation to urological dysfunction will require overcoming some known challenges. First, it needs to be determined if electrical pelvic nerve stimulation can be used to produce predictable therapeutic outcomes. Animal models allow the urodynamic effects of electrical pelvic nerve stimulation to be measured by constant flow cystometry. This assay is based on a method of functional clinical assessment and uses a catheter to fill the bladder and record physiological LUT activity by measuring changes in the intra-vesical pressure. The resulting cystometrogram is used to track micturition cycles comprising repeated episodes of bladder filling followed by the coordinated contraction of the bladder and opening of the urethral rhabdosphincter that expels urine (Andersson et al., 2011). This activity is produced by a peripheral LUT sensorimotor system controlled by a neural control circuit in spinal cord and brain. Previous work in anesthetized animal models has established that pelvic neuromodulation can both facilitate (Andersson et al., 1990; Dalmose et al., 2002; Peh et al., 2018) or inhibit LUT activity (Crook and Lovick, 2017). However, further characterization of the stimulus-response relationship is clearly needed and strategies to limit off-target effects on other pelvic organs that receive input from the pelvic nerve (e.g., lower bowel and sex organs). For clinical translation, it will also be necessary to optimize devices used for pelvic neuromodulation by adapting designs used for large somatic nerves or visceral nerves such as the proximal branches of the vagus (Fallon and Carter, 2016; Horn et al., 2019; Naufel et al., 2020). Another strategy is to design devices that can self-calibrate by using evoked compound action potentials (ECAPs) to adjust electrical stimulus parameters (Bouton and Czura, 2018; Parker, 2018; Parker et al., 2018).

In this study, we demonstrate that a custom four-electrode planar array can be surgically implanted over the distal end of the pelvic nerve and used to produce urodynamic effects by electrical neuromodulation in awake male rats. The array design most commonly used in the clinic has electrodes placed outside the epineurium in a spiral, split-cylinder or folding cuff that surrounds a length of nerve (Guiraud et al., 2016; Larson and Meng, 2020). Cuff electrode designs limit damage associated with invasive inter- and intra-fascicular designs (Naufel et al., 2020) and provide an electrically insulating enclosure which increases the electrical coupling between the nerves and the stimulating electrodes, reducing thresholds. However, cuff electrodes retain the risk of damaging small nerves by constriction, edge abrasion or evoking a foreign body response that affects the physiological properties of nerve firing (Gonzalez-Gonzalez et al., 2018; Lee et al., 2019; Larson and Meng, 2020). We have used a custom planar array that can be positioned adjacent to the pelvic nerve but is anchored to the surrounding tissue. The design using four electrodes also allowed a recording pair to be used for detecting ECAPs generated by stimulation from the alternate electrode pair. The effects of pelvic nerve implantation and stimulation on voiding were assessed using continuous flow cystometry in awake male rats for periods of up to 8 weeks. At the completion of these longer-term chronic studies, immunohistochemical studies on ganglia and spinal tissues were used to assess whether surgical attachment of the array directly injured neural projections through the pelvic nerve. Patterns of immediate early gene (c-Fos) expression in the lumbosacral cord to identify potential activation of nociceptive circuits were also examined.

## Materials and Methods

### Animals

All animal procedures were approved by the Animal Ethics Committees of St. Vincent’s Hospital (Melbourne), the Bionics Institute or University of Melbourne, and complied with the Australian Code for the Care and Use of Animals for Scientific Purposes (National Health and Medical Research Council of Australia). A total of 24 male Sprague-Dawley rats (8–9 weeks, Biomedical Sciences Animal Facility, University of Melbourne; or Animal Resource Centre, Western Australia) were used for experiments. Implanted rats were housed individually with environmental enrichment under a 12 h light/dark cycle with *ad libitum* access to standard chow and water.

### Design of a Custom Planar Four-Electrode Array

**FIGURE 1:**
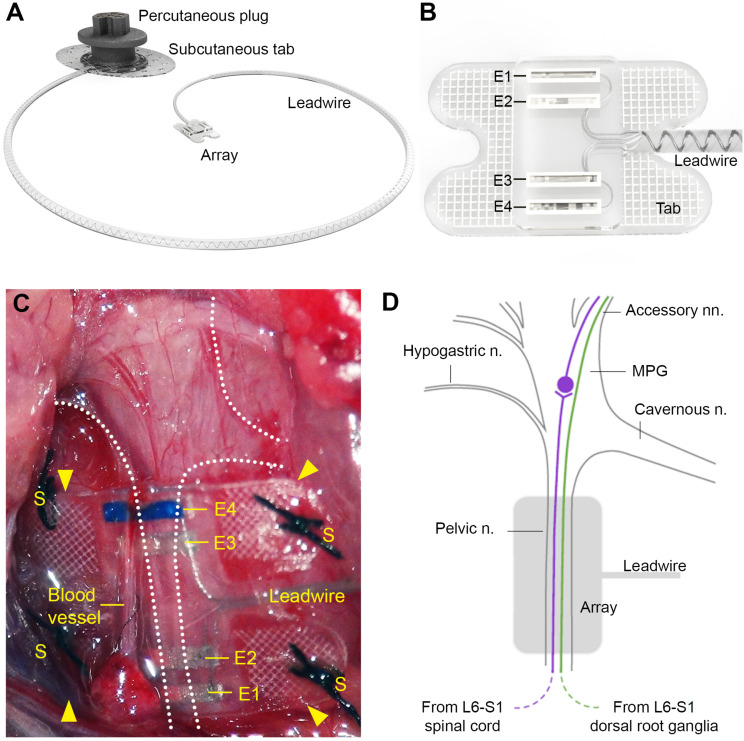
Design of the pelvic nerve electrode array. **(A)** Rendered drawing shows the pelvic nerve array, cable, and percutaneous plug. **(B)** The pelvic nerve array had two platinum electrode pairs (E1–E2, E3–E4) that could be used to stimulate or record evoked neural responses. The array was anchored by suturing the silicon-embedded Dacron tab to connective tissue overlying the prostate gland. **(C)** An *in vivo* image of the implanted pelvic nerve array overlying the right pelvic nerve. Sutures are indicated by “S” and electrodes are indicated by “E1” (closest to the bladder) to “E4” (closest to the spinal cord). **(D)** Schematic diagram indicates the anatomy of the right major pelvic ganglion and its associated nerves, with the location of the electrode array indicated (gray). The pelvic nerve contains parasympathetic preganglionic (motor) axons originating from the intermediolateral column of the L6-S1 cord and sensory axons from the L6-S1 dorsal root ganglia. Preganglionic axons synapse on ganglion neurons that then project to the bladder via the accessory nerves. Sensory axons project to the bladder and colon via the same nerves. Ganglion neurons innervating other pelvic organs are not shown, but include neurons projecting to erectile tissue via the cavernous nerve. Sympathetic ganglion neurons are innervated by spinal preganglionic axons entering the ganglion via the hypogastric nerve (not visible here).

The pelvic nerve electrode array (Figure 1A) consisted of four platinum (99.95%) electrodes embedded in a medical grade silicone elastomer cuff. Individually insulated 50 μm diameter platinum/iridium (90/10) wires were welded to each electrode and formed a helical cable which traversed to a percutaneous connector. Each platinum electrode had an exposed recessed surface area of 0.36 mm^2^ (1.8 mm × 0.2 mm). The distance between adjacent electrodes (E1– E2, or E3–E4, center to center) was 0.75 mm, while the distance between electrode pairs (E1–E2 to E3–E4, center to center) was 2.85 mm (Figure 1B). A Dacron embedded silicone tab surrounded the electrode (Figure 1B) to allow anchoring of the array (using sutures) to connective tissue on the surface of the prostate.

### Surgery

Rats were surgically implanted with a pelvic nerve array and a bladder catheter for use in awake ECAP or urodynamic recordings. All surgical procedures were performed under isoflurane anesthesia (3% for induction and 1.8–2% for maintenance, in 1.5–2 l/min oxygen) and aseptic conditions. Analgesia was provided using buprenorphine (Temgesic, 0.5 mg/kg, s.c.) administered prior to surgery and ∼10 h post-surgery.

### Pelvic Nerve Array

This protocol is described in detail in Fallon et al. (2020). To implant the device, the electrode array attached to the lead wire was first subcutaneously tunneled through a skin incision along the dorsal-lumbar aspect of the spine to exit through a ventral midline abdominal incision. This ventral abdominal incision was used to access the abdominal cavity to gently retract the prostate and reveal the right pelvic ganglia and connecting nerves (Figures 1C,D). The pelvic nerve was then identified and carefully cleared of surrounding fat and connective tissue to allow good contact of the electrode and onto the nerve. Care was taken to identify rectal nerves and to avoid damage to them and blood vessels during clearing of connective tissue and implantation of the array. The array was then positioned against the pelvic nerve close to the pelvic ganglion, so that it was aligned with the nerve running perpendicular across all four electrodes. The four tabs were used to suture (7’0 silk, Ethicon, Somerville, NJ, United States) the array to connective tissue superficial to the prostate. The abdominal cavity was then closed around the lead wire and the muscle and skin sutured closed, after which the rat was rotated to expose the dorsal-lumbar aspect of the spine to allow the percutaneous pedestal to be sutured and skin closed around the plug.

### Bladder Catheter Implantation

This protocol is described in detail in Keast et al. (2020a). After exposing the bladder via a ventral midline abdominal incision, the dome was punctured with an 18G needle to allow insertion of polyethylene catheter (PE-10: od: 0.61 mm × id: 0.28 mm; SteriHealth, VIC, Australia) with a flared end (made by heating the tubing). This was secured with a purse-string suture [sterile monofilament suture II PDS (polydioxanone); Ethicon]. The length of the catheter was then passed through a subcutaneous tunnel and externalized by anchoring to the interscapular skin. The free end of the catheter was sealed to prevent leakage. The abdominal wound was closed using suture, and the skin closed using surgical skin staples (Fine Science Tools, Foster City, CA, United States). In the postsurgical period prior to testing, the catheter was infused with Gentamicin (0.2 ml, 40 mg/ml) for 3 days and then daily with 0.9% saline (0.5 ml) (Keast et al., 2020a).

### Recording and Analysis

#### Electrically Evoked Compound Action Potentials and Impedance Testing

A total of five rats (Animal Resource Centre, Western Australia) were used for end-of-life ECAP recordings under urethane anesthesia (1.2 g/kg subcutaneous, Merck (Sigma-Aldrich, St Louis, MO, United States). All rats were placed on a heated pad and kept hydrated (1 ml sterile physiological saline/100 g) for the duration of the recording, after which all rats were euthanized (300 mg/kg intramuscular Lethobarb; Virbac, Wetherill Park, NSW Australia). In a separate cohort, in-life ECAP recordings were also made in three awake rats chronically implanted with a pelvic nerve electrode array. As described in Fallon and Payne (2020), ECAPs were recorded by stimulating with electrode pair E1–E2 (bipolar stimulation, 100 μs pulse width with 50 μs interphase gap; 10 Hz) and recording with electrode pair E3–E4 (bipolar recording). Two sets of ECAPs (averaged from a total of 50 responses each) were made at currents from 0 to 2 mA in 0.1 mA steps. Recordings were sampled at a rate of 100 kHz and filtered (high pass: 300 Hz; low pass: 5000 Hz; voltage gain 10^2^). The ECAP threshold was defined as the minimum stimulus intensity producing a response amplitude of at least 0.05 μV in both recordings.

During the chronic implantation period, the functionality of electrodes was routinely tested by measuring the common ground impedance of electrodes (Fallon et al., 2009). Biphasic current pulses (25 μs per phase and current of 931 μA) were passed between the electrode of interest and all other implanted electrodes, and the peak voltage at the end of the first phase (V_total_) measured. The V_total_ value was then used to calculate total impedance (*Z*_total_) using Ohm’s law (*Z* = voltage/current).

#### Colonic Pressure Responses During Pelvic Nerve Stimulation

In three urethane anesthetized rats, a balloon-catheter was used to monitor pressure in the colon during pelvic nerve stimulation. The latex balloon and the end of the catheter was inserted 8 cm into the distal colon via the rectum and secured to the tail using tape. Sterile saline was infused via the catheter to inflate the colonic balloon to pressures of about 30–35 mm Hg. Colonic pressure changes (MLT0670, ADInstruments, NSW, Australia) were recorded (Cerebrus, Blackrock, Preston, VIC, Australia) in response to pelvic nerve electrical stimulation delivered at 10 Hz, 100 μs pulse width and current of 1 mA. This stimulation level was confirmed to be supra-threshold for all evoked neural populations by recording ECAPs.

#### Bladder Pressure Responses During Cystometry and Pelvic Nerve Stimulation

In our initial studies performed to establish the surgical procedures (*n* = 12), many of the bladder pressure recordings were challenged by several technical issues, such as grooming and exploratory behaviors of the rat and unstable baseline pressures. For detailed quantitative analyses of voiding parameters, we therefore established a habituation protocol similar to that described in Keast et al. (2020a), i.e., prior to cystometry testing, the animals (*n* = 12) were habituated to the testing environment for 3 consecutive days for 30 min per session. The habituation procedure was also important to reduce effects of these non-voiding related behaviors on c-Fos expression (see below) during the testing period. These habituations and experimental cystometry sessions were confined to the morning to minimize effects of diurnal variation. In these sessions, each rat was placed unrestrained in a clear Perspex box (20.5 × 20.5 × 14 cm) with a mesh floor, elevated on a 45 cm high frame. This allowed free flow of urine during voiding. During habituation periods, the percutaneous plug and catheter were connected, but no stimulation or saline infusion was delivered.

Between 7 and 18 days following the implantation surgery, cystometry and stimulation testing was conducted over a 2-h period. Saline was infused at a rate of 100 μl per minute (HA33, Harvard Apparatus, Holliston, MA, United States) and pressure changes during cystometry testing were transduced (MLT0670, ADInstruments), amplified (PowerLab 4/26, ADInstruments), sampled at 1 kHz (PowerLab, AD Instruments) and viewed using Labchart (AD Instruments). After establishing a stable baseline pressure and inter-void interval (Andersson et al., 2011) the pelvic nerve was stimulated using a custom made external stimulator (Fallon et al., 2018) to deliver 10 s of biphasic current pulses (100 μs pulse width, rate of 10–25 Hz, current levels 0.5–1.5 mA) at selected stages of the micturition cycle. For a subset of experiments, following the cystometry and stimulation testing, each rat was returned to its home cage for 2 h to maximize activity-dependent translation of c-Fos protein (Yap and Greenberg, 2018). Animals were then anesthetized (ketamine: 100 mg/kg and xylazine: 10 mg/kg, intra-peritoneal) and perfused intracardially with fixative, prior to tissue removal for histological study (see below).

Cystometry data were analyzed using customized MATLAB software (R2019b, MathWorks, MA, United States). Voiding contractions induced by continuous flow cystometry were recorded for three cycles prior to the delivery of stimulation and analyzed to determine standard urodynamic parameters (Andersson et al., 2011; Fraser et al., 2020; Table 1). To facilitate comparison with other studies absolute bladder pressures were converted to relative pressures (Table 1) by subtracting the minimum pressure after voiding contractions. High-frequency pressure oscillations (HFPOs), which in rodents are caused by activity of the urethral rhabdosphincter which permits the flow of urine (Andersson et al., 2011; Fraser et al., 2020), detected during voiding contractions were also analyzed to estimate the center frequency of oscillation.

**TABLE 1:** Urodynamic parameters of unstimulated voiding during continuous cystometry in awake male rats after implanting pelvic nerve arrays.

Parameter (units)^a^	Mean (95% CI) *n* = 9 rats^b^	Wiedmann et al., 2020 Mean (95% CI) *n* = 6 rats^b^
Minimum pressure (mmHg)	10.3 (7.4–13.2)	–
Relative threshold pressure (mmHg)	6.6 (4.7–8.4)	4.5 (1.8–7.3)
Relative peak (closing) pressure (mmHg)	51.2 (39.5–63.0)	44. 6 (15.3–74)
Contraction duration (s)	28.9 (17.1–37.5)	37.2 (25.1–49.3)
Inter-void interval (min)	7.6 (5.9–9.4)	8 (5.3–10.6)
HFPO^*c*^ frequency (Hz)	10.7 (8.3–13.0)	–

*^*a*^Andersson et al., 2011; Fraser et al., 2020.^*b*^Cystometry in awake male Sprague-Dawley rats.^*c*^HFPO, high frequency pressure oscillation due to rhabdosphincter activity.*

Bladder pressure responses to stimulation were classified as voiding or non-voiding contractions based on the visible excretion of urine. This was typically accompanied by a period of high frequency bladder pressure oscillation.

### Immunohistochemical Analysis of Nerve Injury Markers

Pelvic ganglia and dorsal root ganglia (L6 and S1), ipsilateral and contralateral to the implant were removed from two rats, 50 days post-implantation and immunostained for neural injury markers. Specifically, these animals were anesthetized (ketamine: 100 mg/kg and xylazine: 10 mg/kg, intra-peritoneal) and perfused intracardially with saline (0.9% sodium chloride, 1% sodium nitrite, and 5000 IU/ml heparin) then fixative (4% paraformaldehyde in 0.1 M phosphate buffer, pH 7.4). The detailed perfusion method has been published (Keast and Osborne, 2019). The dissected ganglia were then post-fixed overnight in the same fixative. Following three 15-min washes (0.1 M phosphate-buffered saline; PBS, pH 7.2), the ganglia were cryoprotected (0.1 M PBS containing 30% sucrose), embedded in an inert mounting medium (OCT; Tissue-Tek, Sakura, Torrance, CA, United States) then sectioned on a cryostat. Serial frozen sections (14 μm) aligned with the major axis of each ganglion were collected onto gelatin-subbed slides. These were washed in PBS and incubated in PBS containing 10% non-immune horse serum and 0.1% Triton X-100. Sections were then incubated for 18–24 h at room temperature with an antibody against: ATF-3 (host rabbit; 1:500, Santa Cruz Biotechnology; sc-188, batch J2209; RRID:AB_2258513); calcitonin gene-related peptide (CGRP) (host goat, 1:2000; AbD Serotec, now Bio-Rad, Gladesville, NSW, Australia; 1720-9007, batch 1705; RRID:AB 2290729; or synaptophysin (host mouse, 1:200; Dako, now Agilent Technologies, Mulgrave, VIC, Australia; M0776, batch 13; RRID:AB_2199013). After washes in PBS, sections were then incubated for 2 h at room temperature with fluorescent secondary antibodies (Jackson Immunoresearch, West Grove, PA, United States): anti-mouse AF488 (1:2000, 715-545-150, RRID:AB_2340846); anti-goat AF594 (1:500, 705-855-147, RRID:AB 2340433); anti-rabbit AF488 (1:1000, 711-545-152, RRID:AB 2313584). Sections were then washed in PBS, mounted onto glass slides, cover-slipped with carbonate-buffered glycerol (pH 8.6) and viewed using a Zeiss AxioImager M2 (Zeiss, Oberkochan, Germany). Sections were assessed qualitatively to determine the features of structures labeled for each of the neural markers. Representative regions of ganglion were imaged to document the primary outcomes.

### Neuronal c-Fos Activity Mapping in Spinal Cord

Neuronal activity mapping was used to detect electrically evoked noxious neuronal activation in lumbosacral spinal cord. After completing the final test session of electrical pelvic nerve stimulation (100 μs pulse width, rate of 10–25 Hz, current levels 0.5–1.5 mA) and cystometry recording on post-surgery day 10, three animals were returned to their home cage for 2 h before being anesthetized and fixed (see above). As described in Keast et al. (2020b), the spinal cord was removed and segments L5-S2 post-fixed for 1 h in the same fixative. Following three 1 h washes (0.1 M phosphate-buffered saline; PBS, pH 7.2), the tissue was cryoprotected (0.1 M PBS containing 30% sucrose), embedded in an inert mounting medium (OCT; Tissue-Tek, Sakura, Torrance, CA, United States) then sectioned on a cryostat. Sections (40 μm) were cut in the transverse plane and collected as four 1:4 series (160 μm between sections), such that five sections per spinal segment were to be investigated for c-Fos expression (see below). Specifically, free-floating sections were washed in 0.1 M PBS (pH 7.2) before being incubated for 2 h in 0.1 M PBS containing 10% non-immune horse serum (NHS; Sigma-Aldrich) and 0.5% Triton X-100. Sections were then incubated for 48–72 h at room temperature with an antibody against c-Fos [1:100; Santa Cruz Biotechnology, Inc., Santa Cruz, CA, United States; (E-8) sc-166940; batch D2318; RSID: AB_10609634]. The c-Fos antibody was diluted in PBS containing 0.1% sodium azide, 2% NHS, and 0.5% Triton X-100. After washes in PBS, sections were then incubated for 4 h at room temperature with Cy3-labeled donkey anti-mouse (Jackson Immunoresearch, West Grove, PA, United States; 715-165-150; batch 89001; RSID: AB_2340813; 1:2000). Sections were then washed in PBS, mounted onto glass slides, and cover-slipped with carbonate-buffered glycerol (pH 8.6).

For each spinal cord segment (L5-S2), the five spinal cord sections were anatomically ordered from rostral to caudal. Entire transverse sections were imaged (tile scanned at 12 Bit, pixel scaling 0.645 μm × 0.645 μm) using an AxioImager M2 microscope and AxioCam monochrome digital camera controlled by Zen software (Zeiss, Oberkochan, Germany). In each section from each of the segments, positive neurons were counted across spinal cord regions, defined by the boundaries described previously (Watson et al., 2009; Figure 5A). For segments L6 and S1, the sacral preganglionic nucleus (SPN) was defined as previously outlined in our earlier study (Forrest et al., 2015). Neurons were counted using ImageJ FIJI Cell Counter plugin, where a marker (denoting the xy coordinate) was designated for each positive cell.

### Statistics

Data reporting on latency and threshold of electrically evoked responses are presented as median ± interquartile range (IQR). Differences between electrically evoked neural thresholds data were not normally distributed, and were therefore statistically evaluated using a (repeated measures) non-parametric Friedman test and Dunn’s *post hoc* test. c-Fos Neuron counts in the spinal cord were analyzed using R Project for Statistical Computing (Version 3.5.2; RRID:SCR_001905) and RStudio (Version 1.1.4; RRID:SCR_000432). Two-sample comparisons were made using exact Welsh two sample *t*-tests to estimate means, 95% CIs and *P*-values. Corrections for multiple testing were made using the Hommel step-up modification of the Bonferroni procedure (Blakesley et al., 2009).

### Figure Preparation

Monochrome images were digitally colorized and the contrast and brightness adjusted to best represent the immunostaining as viewed directly with the microscope. Figures were prepared using Adobe Creative Suite (Adobe Systems, San Jose, CA United States).

## Results

### Electrically Evoked Compound Action Potentials

**FIGURE 2:**
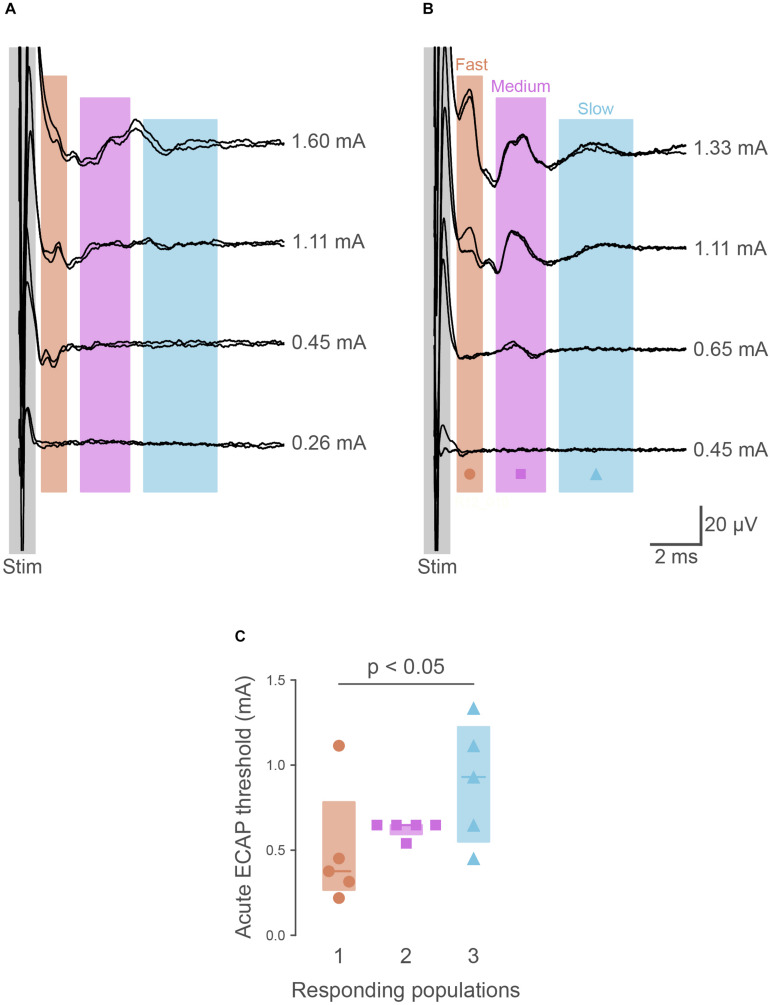
Recordings of electrically evoked neural responses. **(A)** Typically (*n* = 4 of 5), the first responding neural populations had lower thresholds than later responding populations. **(B)** In one animal, the first responding neural fiber population had a higher threshold than the second. **(C)** Quantification of neural thresholds shows the third responding neural population had significantly higher thresholds (*P* = 0.034) than the first responding. The graph shows data from individual rats, median ± interquartile range.

To determine if the pelvic nerve array could stimulate and record ECAPs, devices were unilaterally implanted in five urethane-anesthetized rats. In all cases, ECAPs with complex waveforms could be recorded from the non-stimulating electrode pair during graded electrical stimulation; these ECAPS could be separated from the stimulation artifact (Figures 2A,B). At supra-threshold stimulation levels these waveforms showed positive peaks centered at: P1, 1.80 ms (range 1.65–2.90 ms, *n* = 5); P2, 3.48 ms (2.51–4.16 ms); and P3, 3.77 ms (3.25–5.67 ms). Approximate conduction velocities for these populations are 1.60 m/s (P1, 0.98–1.72 m/s), 0.82 m/s (P2, 0.35–1.13 m/s) and 0.78 m/s (P3, 0.50–0.88 m/s). The mean stimulation threshold of these peaks increased in the order of the first (P1: 377 μA, 267–783 μA), second (P2: 648 μA, 595–648 μA) and third (P3: 930 μA, 550–1225 μA) responding populations (main effect: non-parametric Friedman’s within-subject ANOVA: *P* = 0.039, *n* = 5) (Figure 2C). However, in one rat the threshold of P2 was higher than P1 (Figures 2B,C).

The ability of the pelvic nerve array to stimulate and record evoked neural potentials was evaluated in chronically implanted rats (*n* = 4) following surgery and prior to cystometry testing. Three neural populations, distinguished by their distinct latencies, were consistently recruited in all animals at 0 and 1 week following implantation. The first responding fiber population (P1) had a mean latency of 2.27 ms (range: 1.93–2.65 ms), second responding fibers (P2) had a mean latency of 3.92 ms (range: 3.19–4.44 ms), and the third (P3) had a mean latency of 5.62 ms (range: 4.57–6.72 ms). Approximate conduction velocities for these populations are 1.26 m/s (P1, 1.08–1.48 m/s), 0.73 m/s (P2, 0.64–0.89 m/s) and 0.51 m/s (P3, 0.42–0.62 m/s). Neural thresholds of first (P1, week 0: 391 ± 138 μA, week 1: 643 ± 300 μA), second (P2, week 0: 677 ± 149 μA, week 1: 1051 ± 275 μA) and third (P3, week 0: 713 ± 119 μA, week 1: 1263 ± 333 μA) responding fiber populations significantly increased at 1 week (Friedman test, *P* = 0.039, *n* = 4, Figure 2C).

Electrode impedances were used to monitor the electrical stability and functionality of each electrode in an array following implantation and surgical recovery. The mean electrode impedance prior to implantation) was 4.19 ± 0.17 kΩ (range 3.16–5.03, *n* = 38 electrodes, 10 arrays). On post-surgical day 1, this increased to 7.25 ± 0.79 kΩ (range: 4.54–15.18 kΩ), and further increased after 2 weeks implantation to 12.04 ± 1.15 kΩ (range: 6.24–18.3 kΩ, 38 electrodes in 10 arrays) and 8 weeks implantation to 13.1 ± 2.06 kΩ (8.0–20.2 kΩ, *n* = 8 electrodes, 2 arrays). No short circuits occurred during the implantation period, and only 2 out of 40 electrodes became open circuit (both within 2 weeks of implantation). In experiments where an electrode became open, stimulation of the pelvic nerve could still be delivered through the remaining electrodes in the array.

### Colonic Pressure Changes and Non-urological Effects of Pelvic Nerve Array Stimulation

Effects of electrical pelvic nerve stimulation were determined on colorectal motility in anesthetized rats. Only 1 of 3 animals responded, showing a small increase in colonic pressure of 2.6 ± 0.1 mm Hg (mean ± SEM, 13 within-subject replicate responses) time locked to pelvic nerve stimulation. No evidence of penile erection was detected by visual monitoring during these trials. Stimulation was confirmed to be suprathreshold by recording ECAPs from all three neural populations (P1–P3).

### Urodynamic Effects of Pelvic Nerve Array Implantation and Stimulation in Awake Rats

**FIGURE 3:**
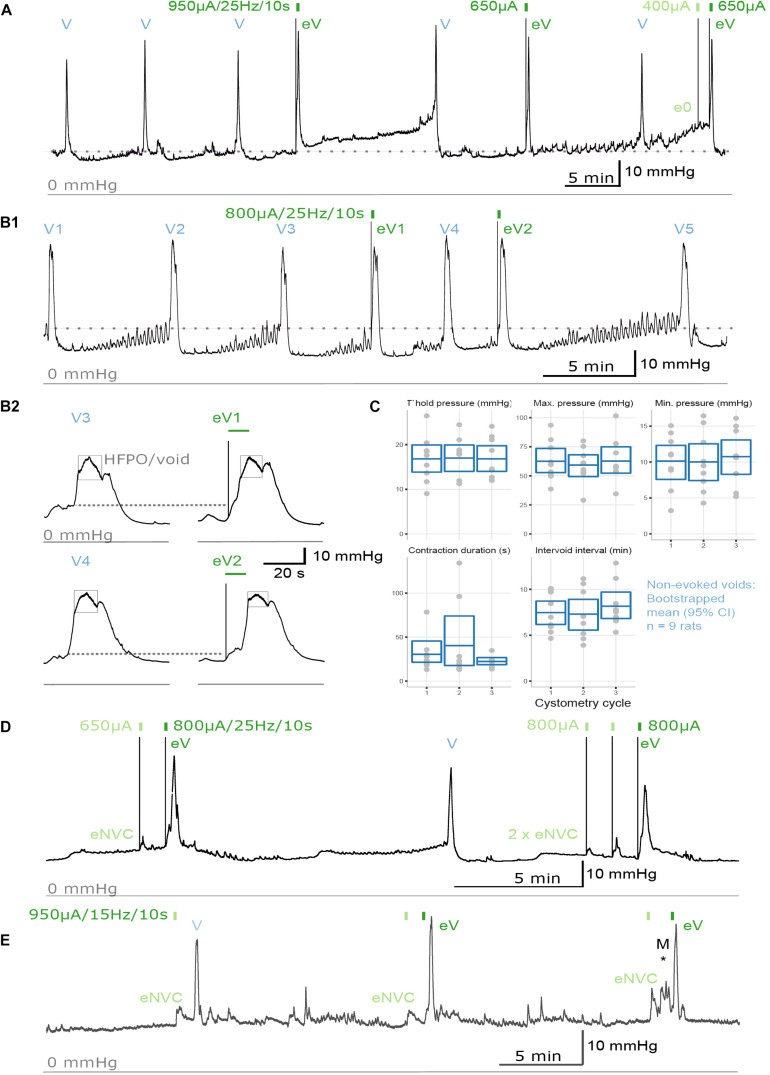
Urodynamic effects of pelvic nerve array implantation and stimulation in awake rats. **(A,B)** Cystometrogram from an awake male rat recorded before and after pelvic nerve stimulation (indicated by rectangles) at 10 days following implantation. **(A,B1)** Implantation of the pelvic nerve array did not affect cystometry-induced voiding (V). Electrical stimulation of the pelvic nerve sometimes evoked urine-producing voids (eV), and rarely caused a null response (e0). **(B2)** Evoked voids were accompanied by characteristic rapid, transient rise in bladder pressure and high frequency oscillations during the period of elevated pressure (eV1, eV2). **(C)** Plots of urodynamic parameters versus cystometry cycle measured in nine awake male rats from contractions recorded prior to any electrical pelvic nerve stimulation. Plotted are data for each subject with boxes showing the mean and 95% CI. **(D)** Electrical stimulation of the pelvic nerve also produced an evoked-non-voiding contraction (eNVC) which resulted in an increase in bladder pressure that was not accompanied with the release of urine. **(E)** At 8 weeks following implantation, stimulation evoked voiding (eV) and non-voiding responses (eNVC) during cystometry. Pressure changes due to animal movement is indicated as “M^*^.”

To study urodynamic effects of the implantation of the pelvic nerve electrode array and stimulation, bladder pressure was measured by continuous-flow cystometry in awake rats (Figures 3A,B). Most recordings were made between 7 and 18 days after surgery, but in two rats recordings were made 8 weeks after surgery (Figure 3E). No obvious adverse behavioral responses to implantation or electrical pelvic nerve stimulation were detected by visual observation of the animals, and stimulation did not cause expulsion of fecal pellets (*n* = 10 animals) or penile erection (*n* = 6). On the day of euthanasia, there were no observed signs of irritation, swelling, infection or adverse reactions to the percutaneous pedestal, lead wire, and electrode array.

The effects of implantation of the pelvic nerve electrode array on filling evoked voiding contractions (*n* = 9) are summarized in Table 1 and Figure 3C. These urodynamic parameters were comparable to published values (Zhao et al., 2010; Andersson et al., 2011; Fraser et al., 2020; Wiedmann et al., 2020). Our priority in assessing the effects of stimulation of the pelvic nerve was to determine the ability of stimulation to evoke a voiding contraction. Therefore, we targeted the majority of our stimulation to occur during a period where the bladder was at least partly filled (estimated as 50–80% based on the time elapsed since the previous filling-evoked void) to ensure the bladder contained sufficient urine for a visible voiding response. Electrical pelvic nerve stimulation was assessed in eight rats in which experimental cystometry sessions were performed following habituation. When delivered during this partly filled state, electrical stimulation of the pelvic nerve was immediately followed by post-stimulation voiding (urine release) in seven of eight rats and was repeated in 3–6 cycles in six of these rats (Figures 3A,B,D). Each void was accompanied by a rapid, transient rise in bladder pressure and high frequency oscillations during this period of elevated pressure that we interpret as indicative of urethral sphincter function characteristic of rodents (Figure 3B2). Given we targeted a partial fill of 50–80%, we consider it unlikely that on each of these occasions we inadvertently chose the precise time for pelvic nerve stimulation that the animal would have normally voided. In support of these being stimulus evoked voiding contractions, in two rats stimulus evoked voiding was even more clearly demonstrated by the pelvic nerve induced void occurring very early in the normal void cycle (Figures 3A,B1).

Electrical stimulation of the pelvic nerve also produced non-voiding bladder pressure responses (not accompanied by release of urine) in six of eight rats (Figure 3D). These non-voiding responses could occur independently, or within the same cystometry session as post-stimulation voiding contractions. In the latter case, it was common to observe stimulus evoked voiding contractions early in the experimental session, but as the session progressed, voiding responses became less frequent and non-voiding pressure changes become more common.

The long-term effects of implantation of the pelvic nerve electrode array and stimulation on bladder pressure was also tested in two animals that had arrays implanted for 8 weeks. In both cases, stimulation produced both voiding (indicated in as “eV” in Figure 3E) and non-voiding responses (indicated in as “eNVC” in Figure 3E) during cystometry (Figure 3E). The urodynamic parameters of filling evoked voids and stimulation evoked voids where similar to those in rats implanted for the shorter duration.

### Post-mortem Analysis of Pathology After Chronic Implantation of Pelvic Nerve Array

On the day of euthanasia, a macroscopic examination of the status of the tissue surrounding the percutaneous connector, cable and pelvic nerve electrode array was conducted. In all of the experimental implanted animals, including the two rats in the 8 weeks recovery group, fibrous tissue surrounding the percutaneous connector and the subcutaneous cable was free from infection and no signs of irritation or inflammation were observed. We also performed a more detailed investigation in the two animals that were implanted for the longest duration (8 weeks). Here, the bladder catheter appeared firmly implanted into the dome of the bladder, and the catheter-bladder entry point had healed well and had no structural or intravesicular irregularities or disruptions. The prostate, seminal vesicles and vas deferens were free from adhesions, infection, inflammation and vascular disruptions. The implanted lobe of the prostate appeared normal and was comparable to the non-implanted, contralateral prostate lobe. A thin fibrous tissue encapsulation had formed around the array further stabilizing the device. The tissue encapsulation was restricted to the vicinity of the pelvic nerve array and did not spread from this area to affect adjacent tissues. Suturing of the electrode array to the soft tissue overlying the prostate had not caused any macroscopic damage to the prostate, pelvic nerve or blood vessels. Furthermore, no irritation, hemorrhaging or hematomas were observed within adjacent tissue.

### Immunohistochemical Analysis of Neural Injury Markers in Ganglia

**FIGURE 4:**
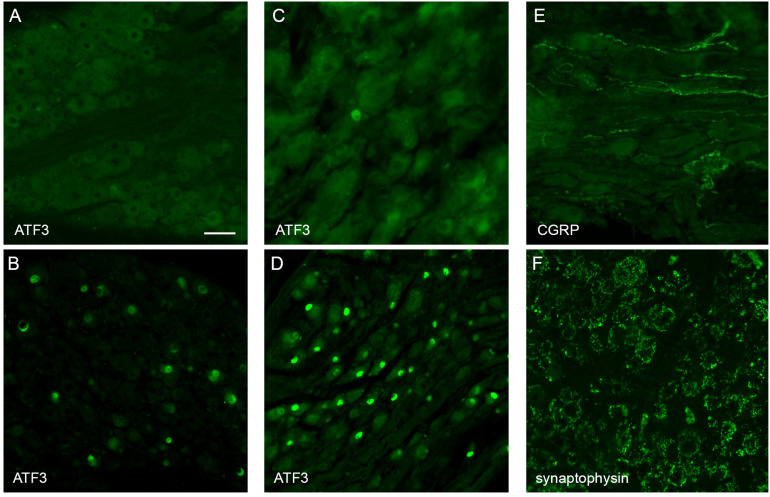
Histological assessment of L6 dorsal root ganglia (DRG) and major pelvic ganglia (MPG) from male rats. Panels **(A,C,E,F)** show ganglia ipsilateral to the array surgery. **(B,D)** are archived ganglia from an earlier axotomy study (Payne et al., 2015), sectioned and immunolabelled as a technical control for the current study; in this earlier study the accessory nerves (mixed sensory-autonomic nerves projecting from the MPG to the bladder) were transected and tissues dissected one week later. **(A)** A DRG ipsilateral to the array surgery shows no ATF3-positive nuclei. **(B)** A DRG from the prior accessory nerve axotomy study shows numerous ATF3-positive neuronal nuclei. **(C)** MPG ipsilateral to the array surgery shows a single ATF3-positive nucleus amongst many neurons with ATF3-negative nuclei. **(D)** The MPG from the prior axotomy study shows numerous ATF3-positive neuronal nuclei. **(E)** In the MPG ipsilateral to array surgery, peptidergic sensory axons traverse the ganglion tissue. **(F)** The MPG ipsilateral to the array surgery shows numerous synaptic boutons immunolabelled for synaptophysin, surrounding each of the MPG neurons. Calibration bar in A represents (μm): A (50 μm), B (50 μm), C (30 μm), D (60 μm), E (30 μm), F (50 μm).

Immunohistochemical visualization of neural markers was performed in ganglia dissected from two rats, 50 days after implantation surgery. Several approaches were chosen to determine if this chronic implantation led to significant neural damage. The pelvic nerve contains the major source of LUT afferent axons, which project from sensory neurons in the lumbosacral (L6 and S1) DRG (Nadelhaft and Booth, 1984). To determine if these axons were damaged by the implanted device we performed immunohistochemistry on these ganglia to detect neuronal expression of the neural injury marker ATF-3 [activating transcription factor-3 (Tsujino et al., 2000; Payne et al., 2015)]. ATF3-positive neuronal nuclei were absent or rare in sections taken from ganglia either ipsi- or contralateral to the implant (Figure 4A). As a positive control, we stained sections from archived DRG in which sacral bladder afferents had been injured by surgical transection of the accessory nerves (mixed sensory-autonomic tracts projecting to the bladder) (Payne et al., 2015). As expected, numerous ATF3-positive neuronal nuclei could be observed in these ganglia (Figure 4B).

The MPG contains most of the autonomic ganglion neurons that project to the pelvic organs, including innervation of the LUT. We considered that the close proximity of the implanted pelvic nerve array to the MPG could potentially injure MPG neurons, either during the surgical implantation procedure or the postsurgical experimental period. However, very few or no ATF3-positive neuronal nuclei (Figure 4C) were seen in sections from MPG ipsi- or contralateral to the array, whereas numerous ATF3-positive nuclei were present in sections of archived MPG from a prior study on accessory nerve injury, used as the positive control (Payne et al., 2015; Figure 4D).

As an alternative approach to assess the impact of the implantation surgery, we examined within the MPG several classes of axons that are known to project in the pelvic nerve and then traverse or terminate in the MPG. First, using a marker of the peptidergic class of sensory axons, CGRP, many axons were observed to traverse both ipsi- and contralateral MPG (Figure 4E). The other major class of axons projecting in the pelvic nerve originates from sacral preganglionic neurons. These parasympathetic pre-motor neurons are essential for the voiding reflex; their axons innervate cholinergic pelvic ganglion neurons, which in turn cause contraction of the bladder muscle (detrusor). Injury to sacral preganglionic axons was examined by assessing the presence of synaptic boutons associated with pelvic ganglion neurons. If preganglionic axons traveling in the pelvic nerve were damaged, these would be lost from the majority of MPG neurons (the other neurons are innervated by lumbar spinal axons traveling in the hypogastric nerve). We found that following 50 days implantation of the device, synaptic boutons innervated the entire population of MPG neurons, ipsilateral and contralateral to the surgery (Figure 4F). Taken together, these observations suggest that implantation surgery did not cause significant damage to preganglionic or sensory axons projecting in the pelvic nerve, or the nearby MPG neurons.

### Immunohistochemical Analysis of Noxious Neuronal Activity in Spinal Cord

**FIGURE 5:**
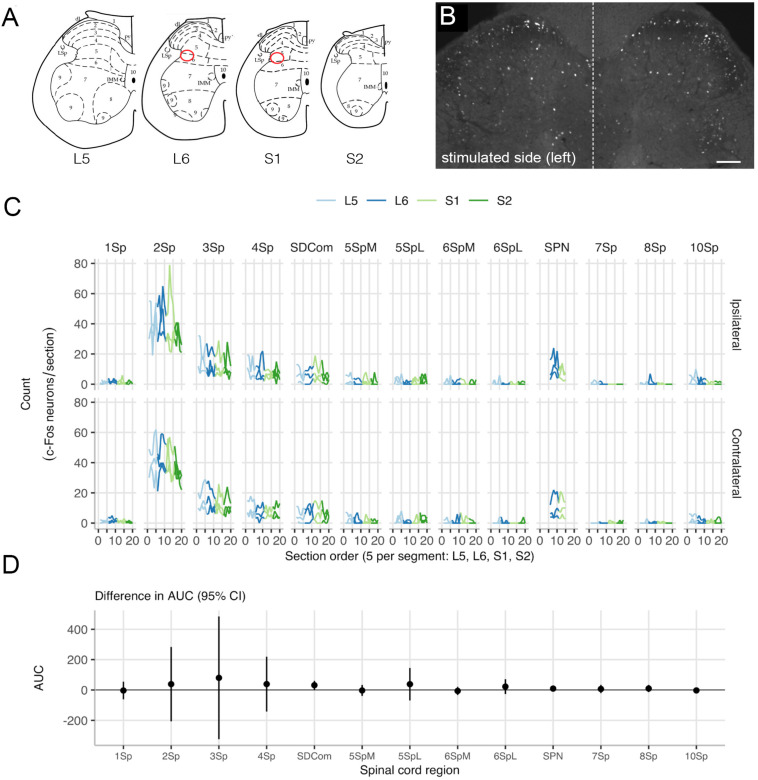
Mapping c-Fos expression in the lumbosacral cord after pelvic nerve stimulation and implantation of an electrode array. **(A)** Expression of c-Fos in neuronal nuclei was mapped in relation to specific spinal regions (Watson et al., 2009). Preganglionic neurons that project in the pelvic nerve are aggregated in the sacral parasympathetic nucleus (SPN), indicated by the red oval. **(B)** An example of c-Fos immunoreactivity showing both sides of an L6 spinal cord section, from a rat where the left pelvic nerve was implanted with an electrode array. This animal underwent regular filling cystometry in addition to activation of the pelvic nerve via the array. **(C)** Spatial analysis of c-Fos-immunoreactive neuron counts in lumbosacral cord shows there were no differences between the left side that received an implant and stimulation, and the right (i.e., control) side. **(D)** Data from each rat is plotted separately (thin lines) as well as the mean for each side (thick line). The region of interest (ROI) neuronal counts by subject show rostrocaudal distribution across spinal cord segments L5 to S2. ROIs: lamina I (1Sp), lamina II (2Sp), lamina III (3Sp), lamina IV (4Sp), sacral dorsal commissural nucleus (SDCom), lamina V (5Sp), lamina VI (6Sp), sacral preganglionic nucleus (SPN), lamina VII (7Sp), lamina VIII (8Sp), and lamina X (10Sp). Scale bar in B represents 100 μm.

Neuronal activity (c-Fos) mapping in sacral spinal cord was used to detect noxious activation caused by the implanted arrays. Changes in c-Fos expression was assessed (*n* = 3) on day 10 of implantation, following 60 min of cystometry and pelvic nerve stimulation testing. In spinal segments L5-S1, neurons with c-Fos^+^ nuclei were most densely distributed in dorsal horn, the sacral dorsal commissural nucleus (SDCom) and the SPN of segments L6 and S1 (Figures 5A–C), consistent with our recent activity mapping study of cystometry in awake male rats (Wiedmann et al., 2020). No difference was detected in the pattern of c-Fos between the two sides of the spinal cord (Figure 5D). No activation of lamina I neurons was detected in either the stimulated side (array implanted on the pelvic nerve) or the contralateral side (Figure 5C).

## Discussion

Stimulation of the pelvic splanchnic nerves may provide therapeutic benefits for a range of LUT conditions. Here we developed an electrode array that interfaced with the rodent homolog of these nerves, the pelvic nerve, without surgical manipulation of the epineurium. For periods of up to 8 weeks following implantation period, our pelvic nerve electrode array recorded electrically evoked neural activity, caused minimal off-target effects to stimulation and induced voiding and non-voiding contractions of the urinary bladder. Furthermore, during long-term implantation our array remained functional and was well tolerated, with no immunohistochemical signs of damage to sacral sensory pathways projecting in the pelvic nerve or adjacent neural tissue of the major pelvic ganglion. Taken together, these findings support that our custom developed pelvic nerve array is an effective and safe design for long-term implantation and stimulation of small autonomic nerves.

The sharp edges and stiffness of cuff electrode designs often evoke a foreign body response and fibrosis, affecting the physiological properties of nerve firing and the stimulation threshold of the electrodes (Loeb and Peck, 1996; Vince et al., 2005; Wodlinger and Durand, 2011; Restaino et al., 2014). Many small visceral nerves, such as the pelvic nerve, have a prevalence of unmyelinated axons and thinner epineurium, increasing their susceptibility to damage (Grill et al., 2009; McCallum et al., 2017; Gonzalez-Gonzalez et al., 2018). Therefore soft, thin, highly flexible cuff arrays made of thiol-ene/acrylate shaped memory polymer have been used for recording spontaneous neural activity in anesthetized rats during cystometry-induced voiding (Gonzalez-Gonzalez et al., 2018). To overcome the challenges posed by interfacing to small, visceral nerves, we developed a silicone based, extraneural electrode array that was placed on top of the pelvic nerve. This approach did not require surgical manipulation of the nerve, nor did the array physically restrict the nerve. The design also allowed for a “one size fits all” approach and could potentially be utilized for the implantation onto other small nerves. Finally, during the 2- and 8-week implantation periods, only 2 out of 40 individual electrodes failed, both due to a break causing an open circuit, suggesting that our electrode design was robust and suitable for chronic use.

Long-term implantation of the pelvic nerve array was well tolerated by the surrounding pelvic organs and implanted neural tissue. No infections or adverse impact to implanted neural or prostate tissue were seen and the foreign body tissue response formed around the array appeared benign and aided in stabilizing the implant, similar to that described previously (Payne et al., 2018). Neural tissue damage induced by the implanted array was minimal, as evidenced by the robust natural and stimulation-induced voiding responses induced by cystometry testing. This was consistent with our immunohistochemical assessment of neural markers, selected to reveal damage to axons projecting in the pelvic nerve. Sensory neurons in sacral dorsal root ganglia detect bladder distension and project to the bladder via the pelvic nerve and the major pelvic ganglion. Ipsilateral to the implanted array, sacral dorsal root ganglion neurons showed negligible expression of the injury marker ATF3, and their CGRP-positive axons were retained in the major pelvic ganglia. The integrity of the bladder motor pathway was also indicated by the retention of synaptic boutons associated with pelvic ganglion neurons. In this ganglion, neurons expressing ATF3 were rare, indicating they were undamaged by the implantation surgery. Together, these observations support our functional assessment that the sensory and motor components of the pelvic nerve remain healthy after implantation of the array, however it is possible that quantitation of axons within these peripheral tissues or the LUT itself would reveal more subtle effects of surgery.

In this study, the spacing between bipolar electrode pairs was a unique design feature that allowed for the recording of three neural populations, distinguished by the latencies of their response. Generally (*n* = 4 of 5 rats), the fastest responding neural population had lower neural thresholds than the slowest responding population, consistent with the size recruitment principle. However, in one animal the first responding neural population showed higher thresholds than the second responding population. This deviation from the size recruitment principle was likely due to the *in vivo* environment, with the electrode-neural distance having a large impact on fiber recruitment. The exact conduction velocity of the neural responses cannot be determined as the precise location of activation is not known, however the conduction velocities of these three neural populations were consistent with that of subpopulations of myelinated A-fiber (1.6–21 m/s) and unmyelinated C-fibers (0.5–1.6 m/s) identified previously in the pelvic nerve (Shea et al., 2000).

Off-target effects can potentially limit therapeutic stimulation delivery, thereby compromising or limiting the effectiveness of stimulation treatment (Waltz, 2016; Payne et al., 2019). Our pelvic nerve electrode array could potentially impact activity of several pelvic organs, as sensory and motor pathways in this nerve innervate the LUT, lower bowel and reproductive organs (Keast, 2006). Our initial study to investigate efficacy of pelvic nerve stimulation on LUT function did not investigate other potential outcomes of stimulation but did observe acute increases in colonic pressure in the minority (*n* = 1 of 3) of rats, but no penile erection. Without more targeted physiological assays, we cannot discount an impact of pelvic nerve stimulation on non-LUT targets or the vasculature. It may also be possible to target pelvic nerve stimulation to particular neural populations, as afferents innervating the LUT have recently been identified as spatially segregated from rectal afferents (Bertrand et al., 2020). Another potential outcome of pelvic nerve stimulation is activation of nociceptive sensory axons projecting to the bladder. We did not identify behaviors indicating pain during electrode activation, and our studies of c-Fos expression did not detect upregulation in lamina 1 of the spinal cord dorsal horn following pelvic nerve stimulation and cystometry testing in awake rats, either ipsi- or contralateral to the implanted array. This pattern of c-Fos upregulation is characteristic of responses to noxious stimuli in the bladder (Birder and de Groat, 1992, 1993; Lanteri-Minet et al., 1995; Kakizaki et al., 1996; Vizzard, 2000). Our observation of c-Fos expression in other regions of the spinal cord demonstrated non-nociceptive pathways typically activated by cystometry, as reported previously (Birder and de Groat, 1992, 1993; Wiedmann et al., 2020).

A major aim of our study was to assess the efficacy of the pelvic nerve array to induce voiding in the awake, unrestrained rat, including in a chronic, post-surgical setting. Two types of LUT activity were observed: voiding and non-voiding contractions. To our knowledge, this is the first study that has demonstrated pelvic nerve stimulation induced voiding in awake, unrestrained rats. Therefore, we felt it critical to use visual confirmation of stimulus induced voiding. This resulted in the requirement of delivering stimulation to a partially full bladder, to ensure the bladder contained sufficient urine for a visible voiding response. We targeted 50–80% full, based on the time elapsed since the previous void, but precise estimates of “typical” voiding cycle duration were difficult to achieve because many rats showed voiding cycles of variable duration. We also considered choosing stimulation times based on bladder pressure rather than duration since last void, but this also had some limitations in animals where there were movement artifacts (rat moving around the cage) or small, unrelated fluctuations in baseline bladder pressure. The variable duration of voiding cycles also makes it possible that some of our stimuli were delivered when the animal would have voided normally, however we consider it unlikely that in the 27 trials in eight rats where we observed a void within 30 s of our stimulation this was always the case. Furthermore, Figures 3A,B1 clearly demonstrate stimulus induced voiding very early in the normal void cycle. In rats where pelvic nerve stimulation was never associated with a void, the most parsimonious explanation is poor surgical placement of the stimulating array. As ECAP recordings were not performed in all animals, we cannot confirm neural activation in these animals. We have not yet conducted studies to determine the mechanism by which the stimulation initiated voiding or non-voiding contractions. To initiate voiding, it is likely that A-δ sensory pathways were activated, initiating the synchronized coordination of autonomic and somatic motor pathways to contract the bladder while relaxing the urethra and urethral rhabdosphincter (de Groat and Yoshimura, 2009). In some experiments, we also identified stimulation parameters that evoked non-voiding contractions. Pelvic nerve-evoked non-voiding contractions of the detrusor muscle have also been reported in anesthetized rats (Crook and Lovick, 2017; Peh et al., 2018), dogs (Andersson et al., 1990) and pigs (Dalmose et al., 2002) and ascribed to activation of sacral parasympathetic pathways based on nerve crush experiments (Dalmose et al., 2002).

Pelvic nerve stimulation induced voiding was achieved up to 8 weeks post-implantation, our chosen experimental endpoint; however, the continued health of the animals at this time indicates that longer periods of implantation are feasible. Therefore, in summary, the present study demonstrates the efficacy and safety of a novel electrode array designed for long-term stimulation of a small visceral nerve and supports the translation of pelvic nerve stimulation as a potential treatment for a range of LUT dysfunctions.

## Data Availability Statement

The raw data supporting the conclusions of this article will be made available by the authors, without undue reservation.

## Ethics Statement

The animal study was reviewed and approved by the Animal Ethics Committees of St. Vincent’s Hospital (Melbourne) Animal Ethics Committees of the University of Melbourne.

## Author Contributions

All authors made substantial, direct, and intellectual contributions to the study and manuscript. SP conceived and designed the experiments, analyzed and interpreted data, and drafted the manuscript. NW, AW, and PS designed and executed experiments. CE analyzed data and prepared Figures 2, 3. PO, JK, and JF conceived and designed research, interpreted data, and revised the manuscript. All authors approved the final version of the manuscript.

## Conflict of Interest

Research reported in this publication received funding from GSK Bioelectronics Innovation Challenge (JF and JK). This funder was not involved in the study design, collection, analysis, interpretation of data, the writing of this article or the decision to submit it for publication. The remaining authors declare that the research was conducted in the absence of any commercial or financial relationships that could be construed as a potential conflict of interest.
